# NEW FINDINGS ON WORK AND FAMILY USING IPUMS DATA

**DOI:** 10.1093/geroni/igz038.2962

**Published:** 2019-11-08

**Authors:** Phyllis Moen, James Raymo

**Affiliations:** 1 University of MInnesota, Minneapolis, Minnesota, United States; 2 University of Wisconsin-Madison, Madison, Wisconsin, United States

## Abstract

The IPUMS Data in Aging Research symposium will showcase aging research that is possible using freely available population-level data accessible via IPUMS. This session will feature papers that use IPUMS data in novel ways to examine aging-related topics including living arrangements, widowhood and divorce, paths toward retirement, intergenerational effects of interventions on later life outcomes, and time use of caregivers. In addition to the common threads of work and family, these papers all represent innovative contributions to aging research based on census and nationally-representative survey data. The potential value of these types of data for studying aging may not be immediately apparent to all researchers. By combining papers on an array of topics from a variety of data sources, this symposium highlights exemplar papers that demonstrate the types of novel research possible using public use census and survey data.

